# Fistulization of Peritoneal Hydatid Cyst to the Gastrointestinal Tract: An Unusual Cause of Subacute Intestinal Obstruction

**DOI:** 10.7759/cureus.5978

**Published:** 2019-10-24

**Authors:** Sarthak Sharma, Khalid Mehmood

**Affiliations:** 1 Radiology, Jammu Healthcare and Diagnostics, Jammu, IND; 2 Gastroenterology and Hepatology, Asia Gastro, Liver and Endoscopy Centre, Jammu, IND

**Keywords:** echinococcosis, peritoneal echinococcosis, intestinal obstruction, hydatidiarrhea, ultrasonography, computed tomography, hydatid cyst, intestinal obstruction due to hydatid cyst

## Abstract

Extrahepatic hydatid disease within the abdomen is uncommon, the most common site being the peritoneum. The alimentary tract is a rare site for hydatid cysts. Fistulization of abdominal hydatid cysts to the bowel lumen is a rare occurrence with few cases reported in existing literature. We report a rare case of fistulization of a peritoneal hydatid cyst to the stomach and duodenum with disseminated peritoneal and retroperitoneal hydatidosis, presenting with features of subacute intestinal obstruction. We briefly review the existing literature and discuss the confounding factors that we encountered during the diagnostic evaluation of this rare phenomenon.

## Introduction

Hydatid disease is endemic in Asia, the Mediterranean and South America [1]. The most common sites of involvement are the liver and lung. Extrahepatic abdominal hydatid disease is less common and may involve myriad sites such as the peritoneal cavity, spleen, pancreas, adrenal, retroperitoneum, ovaries and abdominal wall [2-4]. The occurrence of hydatid cysts within the bowel is exceptional. The intestinal cysts may occur primarily within the bowel, or may arise due to communication of pre-existing extra-intestinal cysts with the alimentary tract [5-7].

We present an unusual case of disseminated peritoneal and retroperitoneal hydatidosis with one of the peritoneal cysts fistulizing to the stomach and duodenum. The patient presented clinically with subacute small bowel obstruction and passage of linear, membrane like structures in stools for which the initial clinico-etiological consideration was bowel ascariasis. However, typical findings on cross-sectional imaging and their correlation with past medical history led us to the diagnosis.

## Case presentation

A 54-year-old male, a shepherd by occupation, presented to us with a history of intermittent colicky pain within the central abdomen associated with episodes of bilious vomiting and progressive abdominal distension for three months. He also reported passage of linear membrane-like structures in the stools for two weeks. There were no other reported symptoms. Past medical history was significant for surgical excision of a hepatic hydatid cyst 20 years ago. An initial clinical evaluation had been carried out at a peripheral health centre, wherein the clinical suspicion was of subacute intestinal obstruction secondary to helminthic infestation of the bowel, possibly ascariasis.

At presentation, the patient was afebrile with stable vital parameters. Clinical examination revealed a distended, non-tender abdomen which was dull to percussion with absent fluid thrill or shifting dullness. A right subcostal incisional scar was evident, consistent with history of prior surgical excision of a liver cyst. Auscultation revealed increased bowel sounds. The rest of the systemic examination was unremarkable.

**Figure 1:**
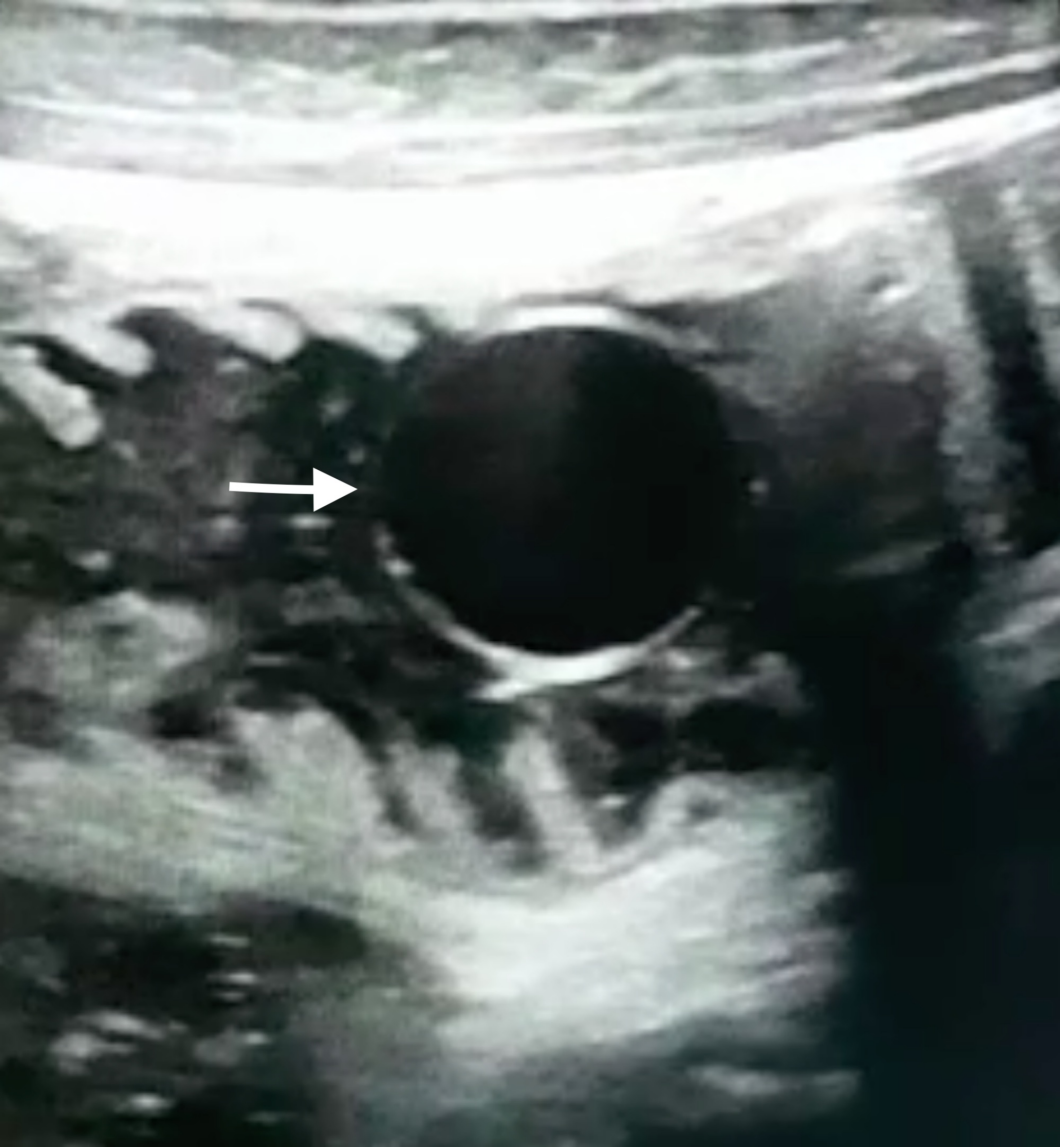
Sonographic image showing intraluminal cyst within a jejunal loop. White arrow depicts the cyst.

**Figure 2:**
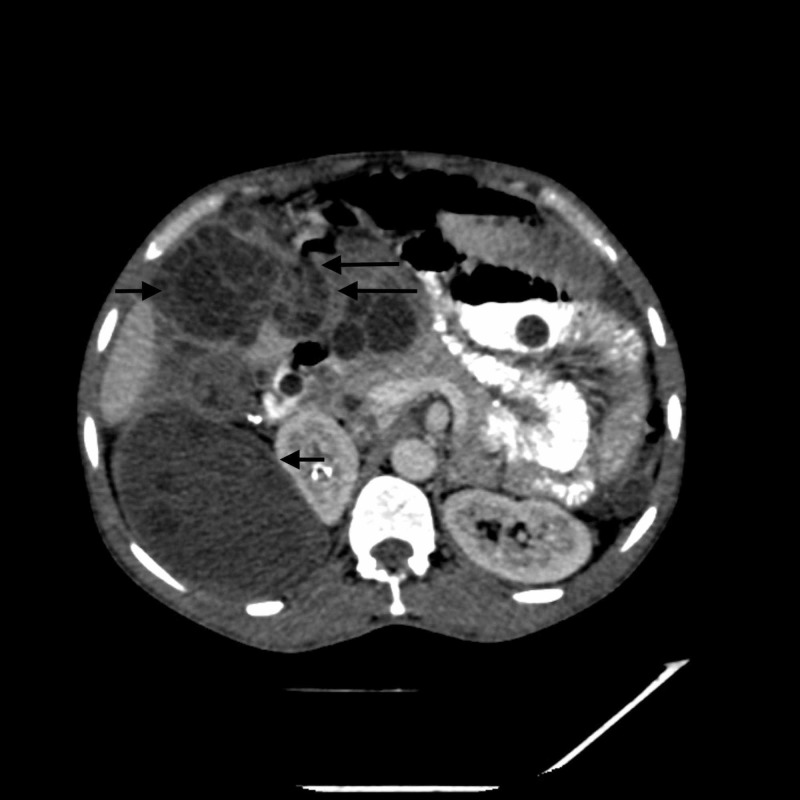
Axial CT image showing communication of peritoneal cyst with distal stomach and proximal duodenum. Long black arrows depict the site of communication. Short black arrows depict peritoneal and retroperitoneal cysts.

**Figure 3:**
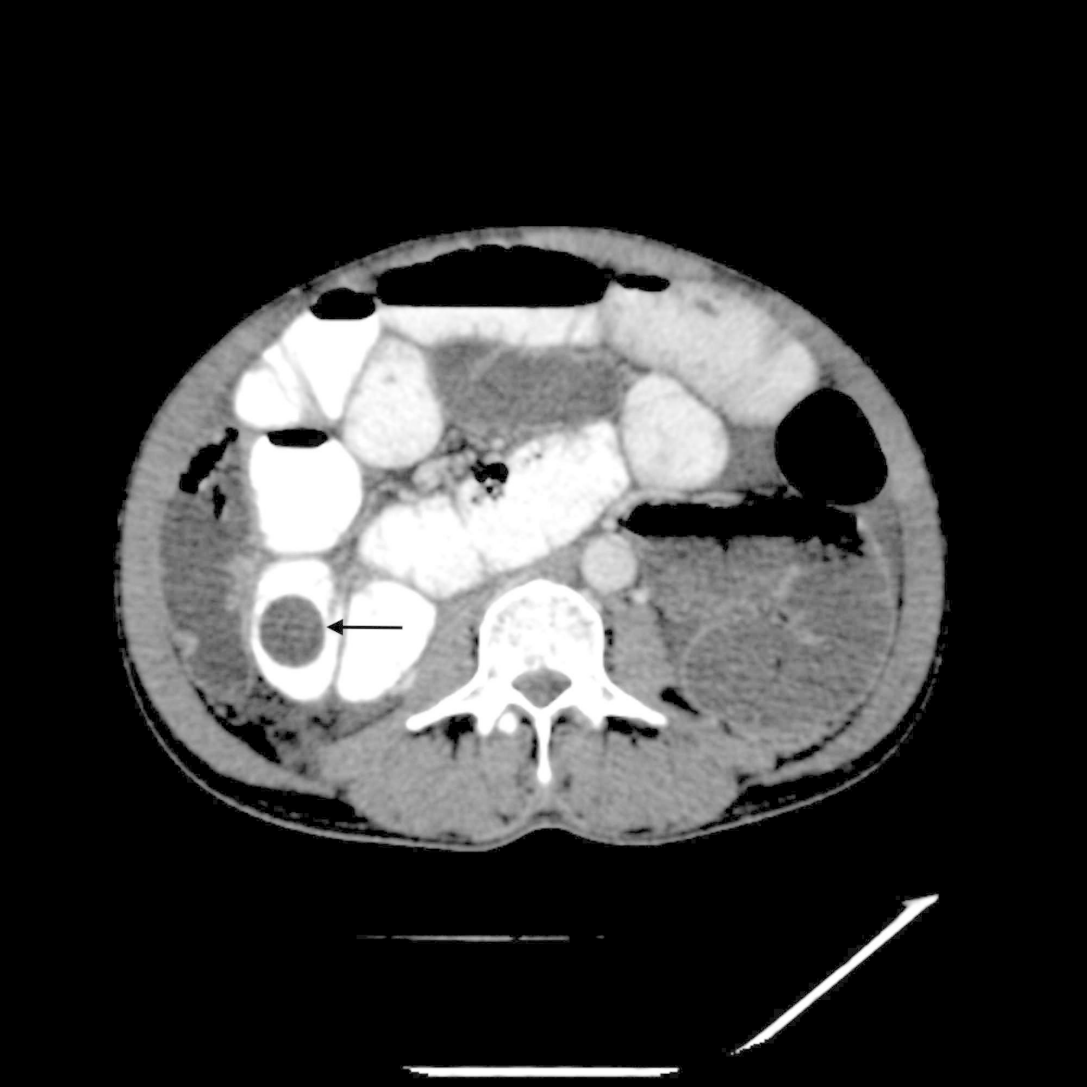
Axial CT image showing cyst within the jejunum. Black arrow depicts cyst within the jejunum.

**Figure 4:**
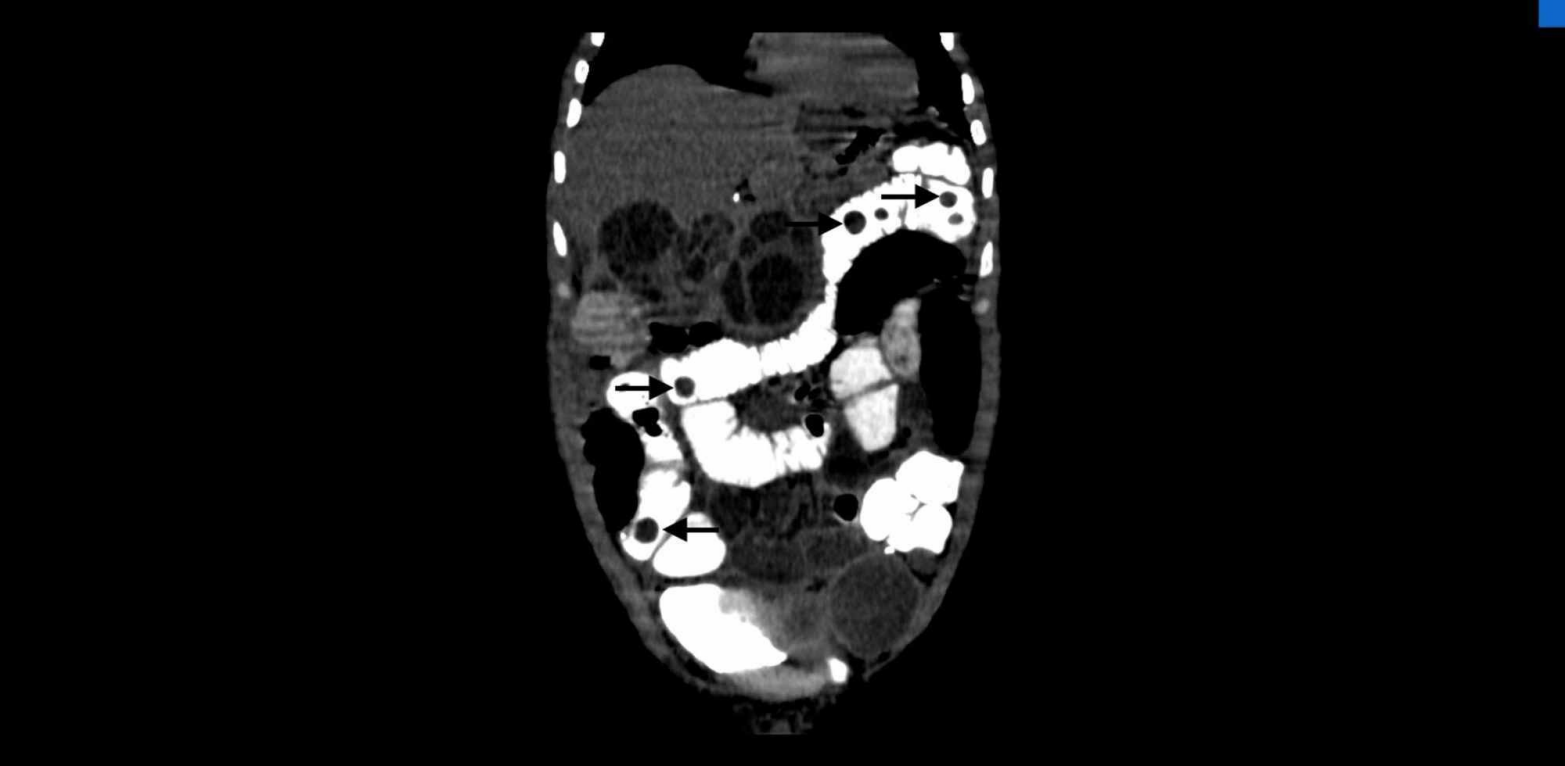
Coronal CT image shows multiple intraluminal cysts within the small bowel. Black arrows depict intraluminal small bowel cysts.

**Figure 5:**
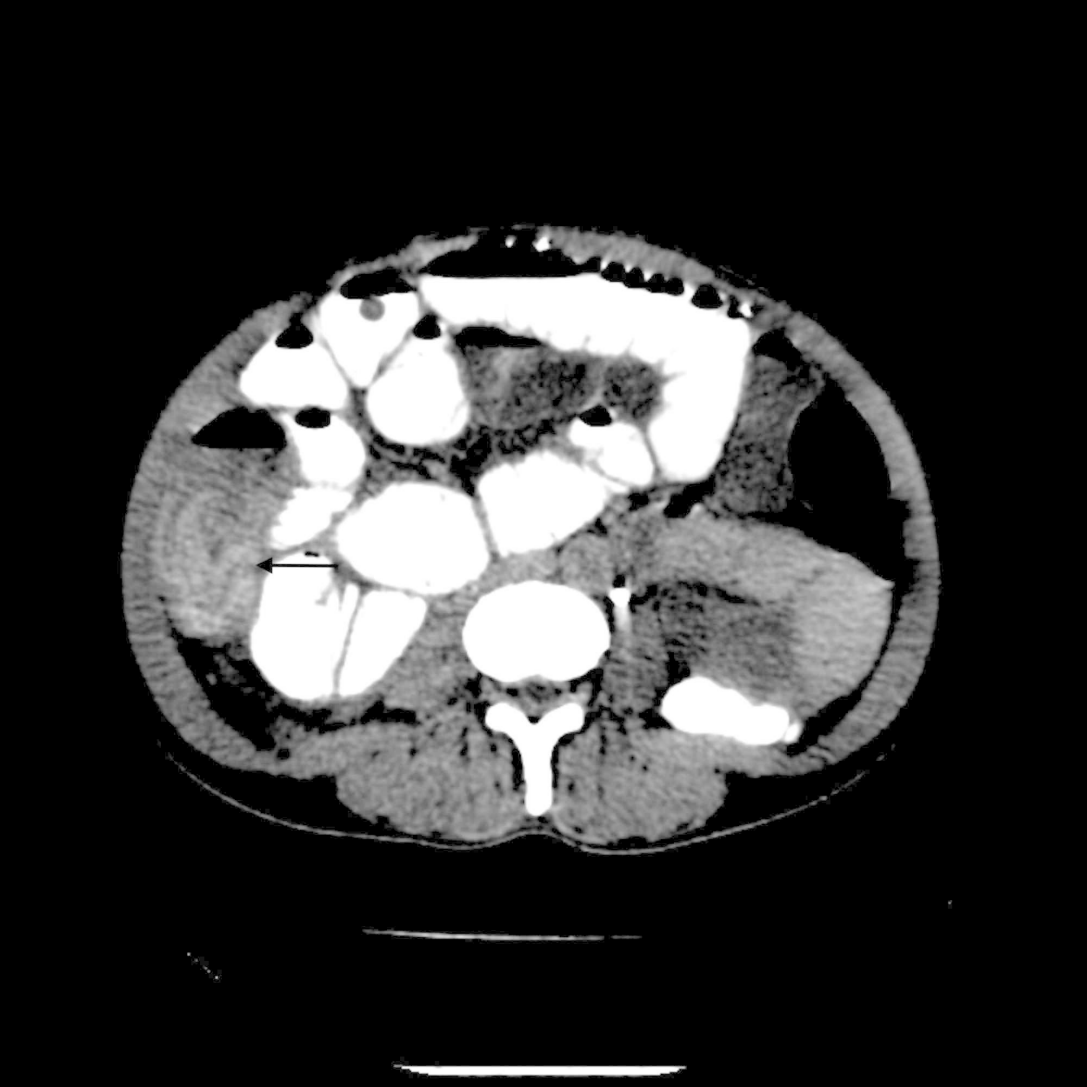
Axial CT image shows membranes within the ascending colon. Black arrow depicts membranes within the colon.

The patient’s hematological and biochemical investigations were unremarkable. Ultrasonography of the abdomen showed multiple cystic lesions within the peritoneum, the retroperitoneum as well as the pelvis, suggestive of hydatidosis. The small bowel loops appeared dilated with exaggerated peristalsis. Some of the small bowel loops within the umbilical region showed multiple small thin walled intraluminal cysts showing to and fro motion with peristalsis (Figure 1, Video 1). Contrast-enhanced CT of the abdomen confirmed the sonographic findings. In addition, CT also showed focal communication of one of the peritoneal cysts with the lumen of distal stomach and proximal duodenum, suggestive of fistulization of the cyst to the alimentary tract (Figure 2). The proximal small bowel loops appeared distended till the level of mid-jejunum. Multiple small intraluminal cysts were seen within the small bowel (Figures 3, 4). The ileum and most of the colonic loops appeared collapsed. Few membrane-like structures, consistent with cyst components, were also seen within the ascending colon (Figure 5).

**Video 1:** Real time ultrasonography shows hydatid cyst within the jejunum moving to and fro with peristalsis.

A diagnosis of transmural gastroduodenal fistulization of a peritoneal hydatid cyst with multiple small intraluminal cysts and membranes within the bowel, subacute small bowel obstruction and concomitant peritoneal and retroperitoneal hydatidosis was made. Echinococcal serological testing, esophagogastroduodenoscopy as well as a colonoscopy were planned but could not be performed in view of the limited financial resources at the patient's disposal. However, typical cross-sectional imaging features and the patient's past history of hydatid liver disease were adequate for making the diagnosis. The patient was started on albendazole therapy and referred to surgical gastroenterology. Following conservative management in the form of nasogastric suction and intravenous fluids for a period of 24 hours, the patient reported marked reduction in the degree of abdominal distension and pain with continuing passage of cyst components in stools. He was counselled regarding the need for surgery, especially the possibility of anaphylaxis. The technical difficulties associated with surgery for disseminated peritoneal and retroperitoneal hydatidosis, as well as the possibility of disease recurrence following surgery were also explained to the patient. The patient refused to undergo surgery, instead opting to continue prescribed medical treatment for a period of four weeks and is under follow-up at the time of writing the report.

## Discussion

Hydatid disease is an endemic zoonosis in Asia, the Mediterranean and South America [1]. The causative organism is *Echinococcus granulosus*. Human beings are accidental intermediate hosts. The commonest sites involved by the disease in human beings are liver and lungs.

Extrahepatic abdominal hydatid disease is uncommon and may involve virtually any site [2-4]. However, the occurrence of hydatid cysts within the bowel is exceptional and scarcely reported in existing literature. The intestinal cyst may present as an abdominal lump, the origin from bowel may be confirmed on cross-sectional imaging [5]. Rarely, primary intraluminal cysts within the bowel may cause chronic intestinal obstruction [6]. In both these instances, the cysts may clinico-radiologically mimic a malignant growth of the bowel and histopathology is essential for making the correct diagnosis.

There are exceptionally few clinical case reports of peritoneal or retroperitoneal hydatid cysts rupturing into the bowel lumen [7-9]. Patients may present with acute abdominal pain and passage of hydatid cysts or membranes in stools as well as vomitus. There may be resolution of a pre-existing abdominal lump following an episode of abdominal colic and passage of hydatid membranes within the stool [8]. Fever, anaphylactoid symptoms and leucocytosis may be accompanying features. The diagnosis may be difficult, especially when the clinical suspicion is low and initial symptoms and clinical investigation results are non-specific and fail to reveal a specific etiology. Cross-sectional imaging and correlation with prior history of hydatid disease are keys to establishing the diagnosis.

In our case, there were a few important differences in clinical presentation as compared to that described in the existing literature. Firstly, the symptoms as reported by the patient were of subacute, rather than acute onset. Secondly, the patient reported no constitutional symptoms, especially fever and presented with stable vital parameters with no anaphylactoid features. Thirdly, the history of progressive abdominal distension, intermittent episodes of colicky abdominal pain and vomiting with passage of linear structures in stools as reported by the patient lead to an initial clinical suspicion of subacute small bowel obstruction due to bowel ascariasis. The presentation was confounded by two more considerations: the patient came from lower socio-economic strata and resided in a geographical region where ascariasis is endemic. It was only upon cross-sectional imaging and correlation with the patient’s remote history of surgical excision of a hepatic hydatid cyst that the diagnosis became clear. This leads us to an important clinical teaching point: the diagnosis of intestinal hydatidosis may be difficult even in areas where hydatid disease is considered endemic.

Treatment options for hydatid cysts of the bowel are surgical as well as medical. Surgical treatment may entail segmental resection of the involved bowel with end to end anastomosis [5]. In cases of associated disseminated peritoneal hydatidosis, the factors influencing selection of medical versus surgical modes of treatment are technical difficulty in clearing the peritoneal disease as well as the possibility of post surgical recurrence. Albendazole is considered the mainstay of medical therapy for hydatid disease. Albendazole therapy may be used in conjunction with surgical treatment, both prior to and after surgery.

## Conclusions

Fistulization of peritoneal hydatid cysts to the alimentary tract is a rare occurrence which may present with features of subacute intestinal obstruction and passage of cyst components in stools. Accompanying constitutional and anaphylactoid features may be absent. The clinical features may significantly overlap with those of bowel ascariasis, which may confound clinical diagnosis. The diagnosis may be difficult, even in areas where hydatid disease is endemic. Correlation with prior history of hydatid disease and cross-sectional imaging is necessary for establishing the diagnosis. The choice of treatment, medical versus surgical, depends upon the expected degree of surgical difficulty and chances of post-surgical recurrence.
